# Development of rat duodenal monolayer model with effective barrier function from rat organoids for ADME assay

**DOI:** 10.1038/s41598-023-39425-7

**Published:** 2023-07-26

**Authors:** Kai Tanaka, Shigeto Kawai, Etsuko Fujii, Masumi Yano, Takashi Miyayama, Kiyotaka Nakano, Kimio Terao, Masami Suzuki

**Affiliations:** 1 Translational Research Division, Chugai Pharmaceutical Co., Ltd., 5-1-1 Tsukiji Chuo-Ku, Tokyo, 104-0045 Japan; 2 Translational Research Division, Chugai Pharmaceutical Co., Ltd., 216 Totsuka Totsuka-Ku Yokohama, Kanagawa, 244-8602 Japan; 3 Translational Research Division, Chugai Pharmaceutical Co., Ltd., 2-1-1 Nihonbashi-Muromachi Chuo-Ku, Tokyo, 103-8324 Japan; 4 Chugai Pharmaceutical Co., Ltd., 1-135 Komakado, Gotemba, Shizuoka 412-8513 Japan

**Keywords:** Gastrointestinal models, Stem-cell differentiation, Intestinal stem cells, Pharmaceutics

## Abstract

The in-depth analysis of the ADME profiles of drug candidates using in vitro models is essential for drug development since a drug’s exposure in humans depends on its ADME properties. In contrast to efforts in developing human in vitro absorption models, only a limited number of studies have explored models using rats, the most frequently used species in in vivo DMPK studies. In this study, we developed a monolayer model with an effective barrier function for ADME assays using rat duodenal organoids as a cell source. At first, we developed rat duodenal organoids according to a previous report, but they were not able to generate a confluent monolayer. Therefore, we modified organoid culture protocols and developed cyst-enriched organoids; these strongly promoted the formation of a confluent monolayer. Furthermore, adding valproic acid to the culture accelerated the differentiation of the monolayer, which possessed an effective barrier function and apicobasal cell polarity. Drug transporter P-gp function as well as CYP3A activity and nuclear receptor function were confirmed in the model. We expect our novel monolayer model to be a useful tool for elucidating drug absorption processes in detail, enabling the development of highly absorbable drugs.

## Introduction

The characterization of absorption, distribution, metabolism, and excretion (ADME) profiles of drug candidates in a preclinical stage is crucial to developing drugs for oral administration because understanding these profiles can help to design the dosage of drug candidates to provide sufficient exposure in in vivo animal models to assess efficacy and toxicity, and to predict drug exposure in humans. In particular, clarifying the absorption profiles of such drugs in the intestinal tract is an important step since intestinal absorption significantly affects drug exposure. Intestinal epithelial cells not only play a role in absorption of nutrients but also form an epithelial barrier through tight-junction and adherence junction proteins which physically and biologically helps prevent invasion by bacteria and penetration of xenobiotics, including drugs. Enterocytes, a major cell type in the intestinal epithelium, expresses not only drug efflux transporters such as P-glycoprotein (P-gp) on the luminal side but also metabolic enzymes, including the Cytochrome P450 (CYP450) family. These key ADME molecules can have a significant impact on drug absorption by blocking drug penetration from the luminal side of the small intestine into the systemic circulation and degrading the drug. Intestinal epithelial cells express nuclear receptors which sense exogenous drugs and endogenous compounds and induce drug transporters and metabolic enzymes. The stimulation of nuclear receptors is known to affect the absorption of drug candidates through alteration of the function of these ADME molecules. Furthermore, there are species differences in CYP-mediated drug metabolism and the induction of ADME key molecules via nuclear receptors^1^.

To evaluate drug absorption in the preclinical stage, in vitro studies with the Caco-2 monolayer model is a de facto standard. Using the human colon adenocarcinoma cell line Caco-2, drug absorption can be evaluated by exposing drug candidates from the apical side of the monolayer model^2^. Although Caco-2 monolayer possess functional drug efflux transporters, this model is not physiologically relevant because it lacks CYP3A4, which accounts for 30% of drug metabolism in humans^3^. To solve this problem, novel intestinal cell sources and culture methods have been developed, such as human primary intestinal epithelial cells^4,5^, modified differentiation protocol of human iPSC-derived intestinal cells^6–10^.

Utilization of intestinal organoids is one of the solutions to address these issues. Organoid culture technology enables to maintain intestinal stem cells in vitro by mimicking the in vivo microenvironment in which intestinal stem cells reside^11^. Intestinal stem cells can be differentiated into absorptive enterocytes, goblet cells, and other types of mature intestinal epithelial cells by modulating culture conditions^12–14^. In contrast to the progress of human in vitro absorption model ^15–19^, a limited number of studies have explored models using rats, which is the most frequently used species in in vivo drug metabolism and pharmacokinetics (DMPK) studies. Intestinal organoid culture methods have been established not only for human intestinal organoids but also for rat duodenal organoids^11,20,21^. Another group reported a method of differentiating rat duodenal organoids and they exhibit drug metabolic activity^22^, but it is difficult to assess drug absorption with organoids since they form closed lumen. The use of a special technique, such as microinjection, can enable access to organoid’s lumen, but such methods are technically difficult and might lack reproducibility and throughput. While rat precision-cut intestinal slices^23–25^ and cell line-derived monolayer models^26,27^ have been reported, there are few reports of luminally accessible monolayer models with functional P-gp, which is encoded by *Abcb1a* and *Abcb1b* in rats. Therefore, a rat monolayer model with transporter and metabolic enzyme functions is required to predict oral drug exposure in rats, to understand the species differences of absorption by comparing them to a human monolayer model, and to accurately predict human absorption.

Here, we developed a luminal accessible monolayer model with an effective barrier function using rat duodenal organoids in an ADME assay. Intestinal crypts were isolated from fresh rat duodenum and embedded in Matrigel to generate rat duodenal organoids, as described by Sato et al.^21^. We modified organoid culture protocols and developed cyst-enriched organoids which generated a confluent monolayer on the cell culture insert. The monolayer culture was successfully differentiated using valproic acid (VPA) while maintaining the barrier function. The established monolayer model showed apicobasal cell polarity, P-gp function, CYP3A activity, and induction capability, and is thus expected to accelerate drug development.

## Results

### Characterization of rat duodenal organoids and evaluation of capability to form confluent monolayer

**Figure 1:**
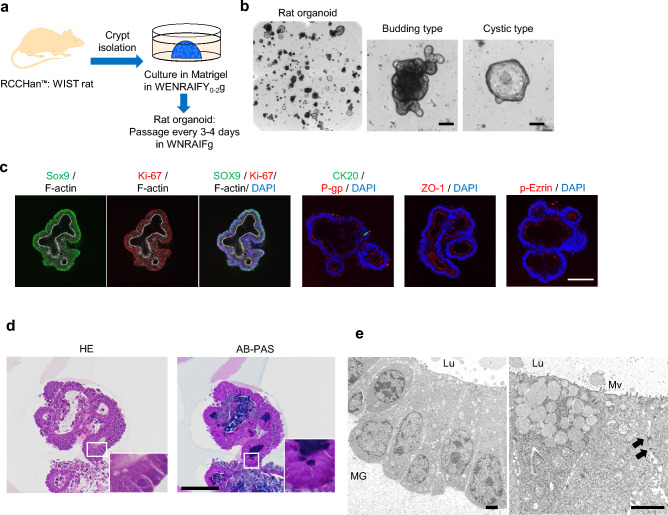
Development and characterization of rat duodenal organoid. (**a**) Scheme of the development of rat duodenal organoids. W, Afamin/Wnt3a CM; E, epidermal growth factor; N, Noggin; R, R-spondin 1; A, A83-01; I, IGF-1; F, FGF-2; Y, Y-27632; g, Gastrin, Y_0–2_ means the supplementation with Y for the first 2 days of culture. (**b**) The bright-field images of organoids. Scale bar = 50 µm. (**c**) Whole mount immunostaining of organoids for Sox9 (green) and F-actin (white), Ki-67 (red) and F-actin (white), merge with DAPI (blue), CK20 (green) and P-gp (red) with DAPI (blue), ZO-1 (red) and DAPI (blue), phospho-Ezrin (p-Ezrin, red) and DAPI (blue). Scale bar = 100 µm (**d**) Day 4 organoids sections were stained with HE and AB-PAS. Scale bar = 100 µm. (**e**) Transmission electron microscopy of organoids on day 4. Arrows, desmosomes; *MG* Matrigel, *Mv* microvilli, *Lu* Lumen. Scale bar = 2.0 µm.

Rat duodenal organoids were established according to a previous report (Fig. 1a)^21^. Organoids mainly consisted of the budding type with a simple columnar shape, but a few of them showed cystic morphology, like a balloon, with an simple squamous shape (Fig. 1b,c). The structural morphology of organoids was similar in the previous report^21^. Organoids expressed stem cell marker SOX9 and proliferative marker Ki67, and the presence of differentiation marker CK20 was also confirmed (Fig. 1c). To characterize them in more detail, pathological analysis and transmission electron microscope analysis were performed (Fig. 1d,e). Organoids encompassed cells stained with alcian blue-periodic acid-Schiff (AB-PAS) and low density granules indicating the presence of goblet cells (Fig. 1d,e). Moreover, tight junction marker ZO-1, drug transporter P-gp, and microvilli marker p-Ezrin were expressed on the luminal side of the organoids. Furthermore, microvilli and tight-junction were confirmed by transmission electron microscopy (Fig. 1c,e). A few of the rat organoids were of the cystic type, which exhibited similar characteristics to that of the budding type (Supplementary Fig. S1a–c). These results indicated that rat duodenal organoids were composed of both undifferentiated and differentiated cells. To develop a monolayer model for an ADME assay, organoids were digested and dissociated into single cells and cultured on cell culture insert, but the adhesion and proliferation of cells were poor and a confluent monolayer did not form until Day 11 (Supplementary Fig. S2). This result suggested that rat organoids, as a cell source, were not suitable for generating a fully confluent monolayer with barrier function, thus improvement was required to overcome this issue.

### Development and characterization of a cyst-enriched organoid culture

**Figure 2:**
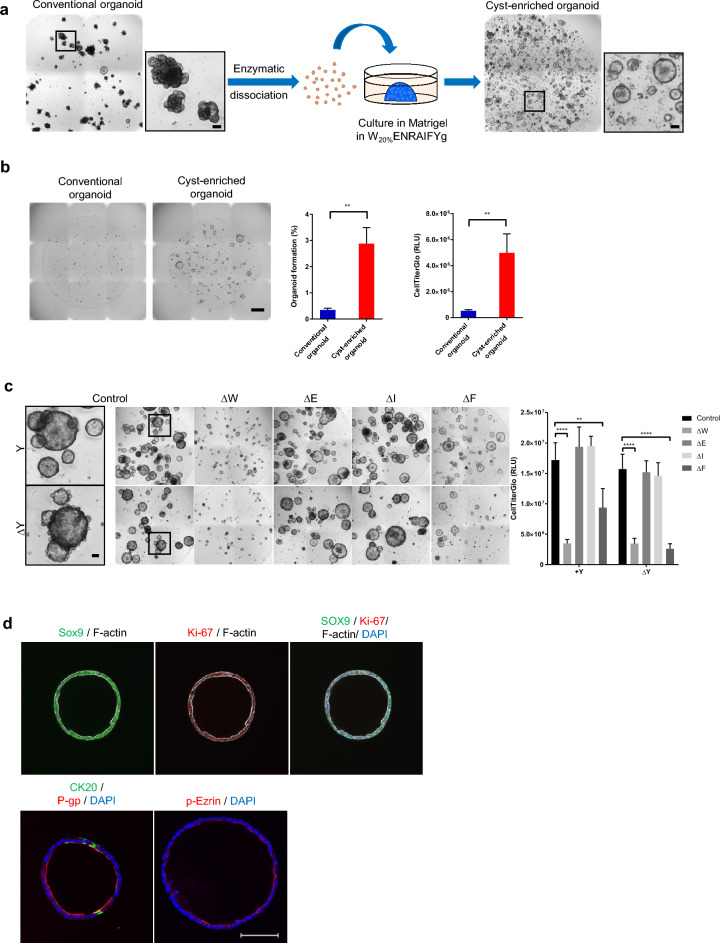
Development and characterization of a cyst enriched-organoid. (**a**) Scheme of the development of a cyst enriched-organoid and the bright-field images of organoids. Scale bar = 100 µm. (**b**) The bright-field images of organoids derived from single cells prepared by day 4 conventional organoids and cyst-enriched organoids. Growth of the organoids was analyzed with CellTiter-Glo^®^ 3D Cell Viability Assay and the bright-field image analysis by cellSens. Data represents mean ± s.d. (n = 3, biological replicates). Scale bar = 1 mm (**c**) The bright-field images of organoids in the growth factor reduced conditions and the results of the growth factor dependency analysis using CellTiter-Glo^®^ 3D Cell Viability Assay. Scale bar = 100 µm. Data represents mean ± s.d. (n = 4, biological replicates). Δ means omitting a specific growth factor from the complete medium. (**d**) Whole mount immunostaining of cyst-enriched organoid for Sox9 (green) and F-actin (white), Ki-67 (red) and F-actin (white), merge with DAPI (blue), CK20 (green) and P-gp (red) with DAPI (blue), phospho-Ezrin (p-Ezrin, red) and DAPI (blue). Scale bar = 100 µm. **p < 0.01, ****p < 0.0001. Student *t* test for organoid formation assay, and Dunnett’s test compared with control for growth factor dependency analysis. *W*_*20%*_ 20% Afamin/Wnt3a CM, *E* epidermal growth factor, *N* Noggin, *R* R-spondin 1, *A* A83-01, *I* IGF-1, *F* FGF-2, *Y* Y-27632, *g* Gastrin.

We speculated that one of the reasons the confluent monolayer did not form might be the high stress, such as the detachment from Matrigel, the extracellular matrix for organoid culture, and the dissociation into single cells, slowed down in proliferation ability and made it easier to cause program cell death of cells prepared from organoids. Indeed, disruption of the interaction between epithelial cells and extracellular matrix induces programmed cell death by anoikis and isolated intestinal crypts rapidly undergo anoikis^28,29^. Therefore, we attempted to modify the organoid culture conditions to enrich organoids which able to tolerate these stress. We designed the organoids into single cells, embedded them in Matrigel, added ROCK inhibitor Y-27632 and EGF to the culture medium, and increased the concentration of Afamin/Wnt3a CM from 10 to 20%. Each of these factors has been reported to prevent anoikis, promote organoid formation, and enhance the Wnt signal which factor is critical to generating organoids and the maintenance of intestinal stem cells in in vitro^11,20,30–32^. As a result, the organoids with cystic morphology were enriched (Fig. 2a). Cyst-enriched organoids showed stable cystic morphology until at least the 28th passage (Supplementary Fig. S3). In order to confirm whether single cells prepared from cyst-enriched organoids show the resistance to anoikis and form organoids, the efficiency of organoid formation from single cells was evaluated using bright-field images and CellTiter-Glo^®^ 3D Cell Viability Assay, which can determine the number of viable cells based on the ATP quantification. As a result, single cells prepared from cyst-enriched organoids formed organoids more efficiently than single cells prepared from conventional organoids (Fig. 2b). This result indicates that cyst-enriched organoids have higher stress tolerance to anoikis.

Generally, the conversion from the budding type to the cystic type was triggered by the overall response to Wnt signaling, which suppresses cell differentiation and transforms the budding organoid consisting with heterogenous cell types into the cystic organoid consisting with more uniform proliferative undifferentiated cells^33^. On the other hand, the cystic morphology of Wnt pathway independent intestinal organoids has already been described in previous reports^34,35^. Therefore, we investigated the dependency on Wnt3a as well as on growth factors and Y-27632 (Fig. 2c). Our results showed that Wnt3a was indispensable for the growth of cyst-enriched organoids (Fig. 2c). Withdrawing Y-27632 induced bud-like structures on the surfaces of organoids, and the removal of both Y-27632 and FGF-2 attenuated organoid growth significantly (Fig. 2c). Therefore, Wnt3a, FGF-2, and Y-27632 are critical components for culturing cyst-enriched organoids. Whole mount immunostaining identified SOX9 and Ki-67 and confirmed CK20 positive cells, and polarized expression of P-gp and p-Ezrin which was similar in conventional organoids (Figs. 1c, 2d).

**Figure 3:**
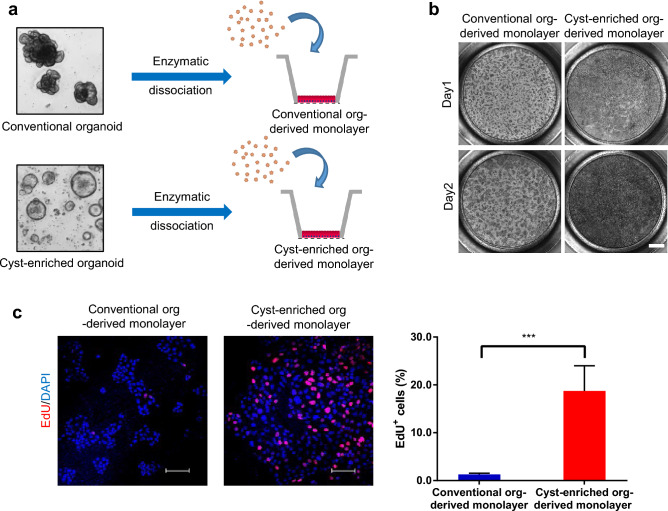
Cyst-enriched organoid are suitable for monolayer formation. (**a**) Scheme of generating the monolayer culture from conventional organoids and cyst-enriched organoids. Day3 or 4 organoids were dissociated into single cells and seeded on Matrigel-coated Transwell (**b**) The bright-field images of monolayer culture derived from single cells prepared by conventional organoids and cyst-enriched organoids. Cells were stained with basal medium containing 10 µM Y-27632 and 0.5 mg/ml MTT. Scale bar = 1 mm (**c**) Results of EdU uptake assay of day 1 monolayer culture from single cells prepared by conventional organoids and cyst-enriched organoids. Monolayer cultures were incubated with expansion medium containing 20 µM EdU for 1 h. Scale bar = 100 µm. Data represents mean ± s.d. (n = 4, biological replicates). ***p < 0.001, Student *t* test. *Org* Organoid.

Next, we determined the capability of the cyst-enriched organoids to form the monolayer on the cell culture insert (Fig. 3a). In stark contrast to the conventional organoids, cyst-enriched organoids accelerated the propagation of the monolayer and reached confluency on day 2 (Fig. 3b). EdU is a nucleoside analog of thymidine and the EdU uptake into nucleus directly represents the active DNA synthesis for cell division. An EdU uptake assay demonstrated that EdU positive cells were significantly increased in the cyst-enriched organoid-derived monolayer (Fig. 3c). Taken together, these results show that we successfully generated cyst-enriched organoids which can tolerate the process of single cell preparation, thus enabling the formation of a confluent monolayer in a short culture period.

### Development of a differentiated monolayer model with effective barrier function

**Figure 4:**
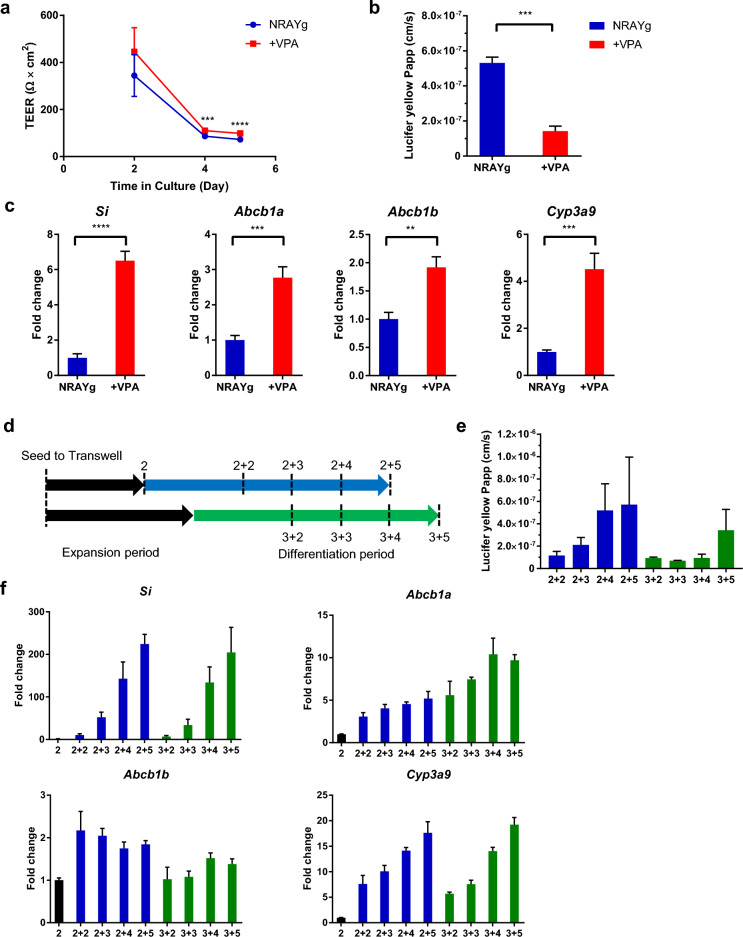
Development of monolayer model using cyst-enriched organoid as a cell source. (**a**) Plots of the TEER values for each day in culture. Differentiation was initiated from day 2. TEER values were corrected by their corresponding background well and area of insert membrane. (**b**) Papp of lucifer yellow in monolayer cultured with expansion medium for 2 days and differentiation medium with and without 3.0 mM VPA for 3 days. Data represents mean ± s.d. (n = 3, biological replicates). (**c**) Expression of differentiation marker (*Si*) and transporter and metabolic enzyme (*Abcb1a, Abcb1b, and Cyp3a9*) in monolayer culture with expansion medium for 2 days and differentiation medium with and without 3.0 mM VPA for 3 days. Data represents mean ± s.d. (n = 3, biological replicates). (**d**) Scheme of evaluation for expansion and differentiation periods. (**e**) Papp of lucifer yellow in monolayer culture with expansion medium for 2 and 3 days and differentiation medium for 2, 3, 4, and 5 days. Data represents mean ± s.d. (n = 3, biological replicates). (**f**) Expression of differentiation marker (*Si*) and transporter and metabolic enzyme (*Abcb1a, Abcb1b,* and *Cyp3a9*) in monolayer culture with expansion medium for 2 and 3 days and differentiation medium for 2, 3, 4, and 5 days. Data represents mean ± s.d. (n = 3, biological replicates). **p < 0.01, ***p < 0.001, ****p < 0.0001, Student *t* test. *N* Noggin, *R* R-spondin 1, *A* A83-01, *Y* Y-27632, *g* Gastrin, *VPA* valproic acid, *TEER* transepithelial electrical resistance, *Papp* apparent permeability coefficient.

To generate a differentiated monolayer model with an effective barrier function and the activity of key ADME molecules, we investigated the differentiation medium and optimal differentiation period considering the balance of maturation and barrier function. VPA, a histone deacetylase inhibitor, has been reported as a Notch signaling activator^36,37^. Notch signaling is dispensable for the differentiation of absorptive enterocytes^38^. After the confluent monolayer formation on day 2, monolayer culture was exposed to VPA combined with the removal of Wnt3a and growth factors. VPA increased transepithelial electrical resistance (TEER) value, which represents the integrity of cellular barrier, and decreased lucifer yellow permeability, the robust paracellular permeability marker, indicating the enhancement of epithelial barrier formation (Fig. 4a,b). Moreover, the expression of not only the enterocyte maker *Sucrase-isomaltase* (*Si*) but also the drug transporter *Abcb1*, *Abcb1b* and metabolic enzyme *Cyp3a9*, the main CYP isoform expressed in rat intestine, were augmented by the administration of VPA (Fig. 4c), therefore we decided to add VPA to the differentiation medium.

Next, the barrier function and gene expression profiles of the monolayer culture were investigated to determine the appropriate combination of expansion and differentiation periods. We combined a 2 and 3 day expansion period with a 2–5 day differentiation period, performed a lucifer yellow permeability assay, and evaluated the gene expression of *Si*, *Cyp3a9,* and *Abcb1a*, *Abcb1b* (Fig. 4d). As a result, the expression level of *Si* and *Cyp3a9* increased over the differentiation period while the barrier function decreased, especially with the 2 day expansion period (Fig. 4e,f). *Abcb1a* showed the same tendency as *Si* and *Cyp3a9*, however *Abcb1b* was not clearly correlated with the differentiation period. In the 3 day expansion, effective barrier function was maintained with the increase of *Si*, *Cyp3a9,* and *Abcb1a* expression (Fig. 4e,f). We concluded that the combination of a 3 day expansion period and a 3 or 4 day differentiation period was better for achieving a balance between barrier function and differentiation.

### Characterization of rat duodenal organoid-derived monolayer model for ADME assay

**Figure 5:**
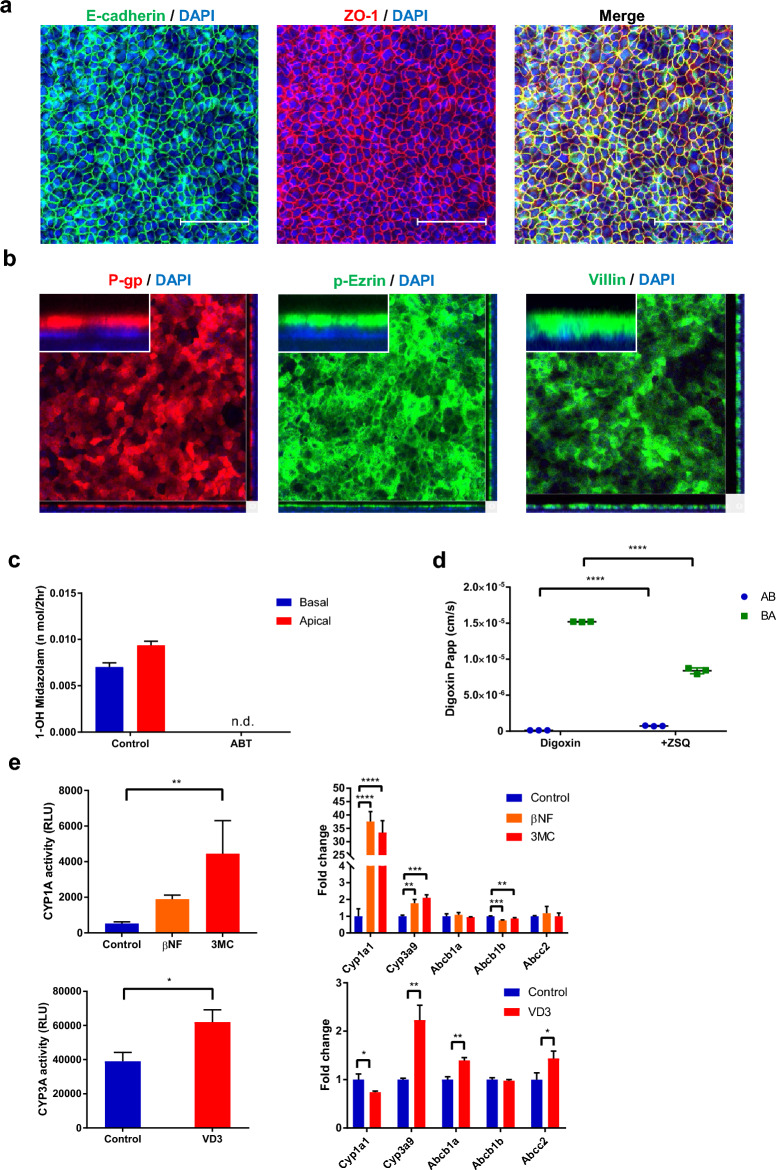
Characterization of monolayer model for ADME assays. (**a**) Whole mount immunostaining of differentiated monolayer culture which stained with E-cadherin (green), ZO-1 (red), F-actin (white) with DAPI (blue). Scale bar = 100 µm (**b**) Whole mount immunostaining of differentiated monolayer culture stained with P-gp (red), phospho-Ezrin (green), or villin (green) together with DAPI (blue). (**c**) Production of 1-hydroxymidazolam with and without 1 mM ABT in differentiated monolayer with 48 h exposure of 100 nM VD3. (**d**) Transporter assay using P-gp substrate 10 µM digoxin which was exposed from apical or basal side of monolayer culture with and without 5 µM Zosuquidar (ZSQ). (**e**) CYP1A activity and CYP3A activity were analyzed by P450-Glo Assays. Expression of *Cyp1a1*, *Cyp3a9*, *Abcb1a*, *Abcb1b*, *Abcc2* were evaluated by RT-PCR. Data represents mean ± s.d. (n = 3, biological replicates). *p < 0.05, **p < 0.01, ***p < 0.001, ****p < 0.0001, Dunnett’s test compared with the control for CYP1A induction assay, Student *t* test for digoxin transport assay and CYP3A induction assay. *Papp* apparent permeability coefficient, *ADME* absorption, distribution, metabolism, and excretion, *ABT* 1-aminobenzotriazole, *ZSQ* zosuquidar, *AB* apical to basal, *BA* basal to apical, *βNF* beta-naphthoflavone, *3MC* 3-methylcholanthrene, *VD3* 1alpha, 25-dihydroxyvitamin D3.

To characterize the suitability of the established monolayer model for an ADME assay, the cell polarity, CYP3A function, transporter function, and nuclear receptor function were evaluated (Fig. 5). Tight junction marker ZO-1 and adherence junction marker E-cadherin were localized to the plasma membrane at sites of cell–cell contact. This resembles the intestinal epithelium and indicates tight barrier formation (Fig. 5a). Not only p-Ezrin and Villin, which are components of intestinal microvilli, but also the drug transporter P-gp were detected at the apical membrane of the differentiated monolayer (Fig. 5b). The results of a metabolic assay with CYP3A substrate midazolam and CYP3A inhibitor 1-aminobenzotriazole (ABT) indicated the CYP3A activity in the differentiated monolayer because midazolam metabolite 1-hydroxymidazolam was present in both the apical and basal compartment, and ABT inhibited the metabolite production (Fig. 5c). Next we conducted an efflux transport assay with digoxin, a well-known P-gp substrate, and the P-gp inhibitor zosuquidar^39^. The efflux transport of digoxin was observed in the absence of zosuquidar. The efflux was attenuated, and influx was increased by P-gp inhibition. This result demonstrated the polarized presence of P-gp protein and functions emulating the intestinal epithelium (Fig. 5d). Finally, the nuclear receptor function was assessed by aryl hydrocarbon receptor (AhR) agonist β-Naphthoflavone (βNF) and 3-methylcholanthrene (3MC), and by endogenous vitamin D receptor (VDR) agonist 1α, 25-dihydroxyvitamin D3 (VD3). βNF and 3MC elevates *Cyp1a1* gene expression and CYP1A enzyme activity in rat intestine through AhR^40^ and VD3 increases *Cyp3a* gene expression and CYP3A enzyme activity through VDR^41^. *Cyp1a1* and *Cyp3a9* were selected as induction monitor CYP genes based on its high expression in rat intestine^42–44^. We treated the monolayer model with βNF, 3MC and VD3 for 48 h and measured the activity and gene expression of CYP1A or CYP3A (Fig. 5e). CYP1A activity was increased by the exposure of βNF and 3MC along with the drastic augmentation of *Cyp1a1* gene expression (Fig. 5e). *Cyp3a9* was increased and *Abcb1b* was slightly downregulated by βNF and 3MC (Fig. 5e). VD3 induced not only the CYP3A activity but also the gene expression of *Cyp3a9, Abcb1a,* and *Abcc2*. *Cyp1a1* was slightly downregulated by VD3 (Fig. 5e). These results confirmed the presence of the functional AhR and VDR in the differentiated monolayer and recapitulated the in vivo response by exogenous and endogenous compounds.

## Discussion

In this study, we developed an apically accessible monolayer model using cyst-enriched organoids as a cell source. The monolayer showed a tight barrier function and possessed characteristics essential for ADME assays that are used to evaluate drug absorption.

Intestinal organoids not only recapitulate the structure and physiology of intestinal epithelium but can also be a useful cell source for generating new in vitro models since they can be expanded for long periods, e.g., 6 months or more for human intestinal organoids^20^. Rat organoids prepared according to a previous report were passaged many times, and the cyst-enriched organoids we established in this study were also maintained for at least 28 passages and could be expanded three- or four-fold every 3 to 4 days. This means that many cells can be prepared from the same donor. Single cells prepared from cyst-enriched organoids grew on cell culture insert rapidly and formed a fully confluent monolayer compared with conventional rat organoids. Therefore, the use of cyst-enriched organoids as a cell source can significantly improve the throughput and reduce the turn-around time of experiments.

To develop a model for assessing drug absorption, it is important to consider not only the barrier function but also the transporter and metabolic functions. A previous study demonstrated that Notch signaling is related to enterocyte differentiation from intestinal stem cells in normal intestine^45^. The combination of Wnt3a/growth factor removal and activation of Notch signaling by VPA treatment successfully induced the expression of enterocyte marker *Si,* consistent with previous report^12^. Moreover, VPA had a positive effect on the development of monolayer culture in that it enhanced the epithelial barrier function and increased the gene expression of drug transporters and metabolic enzymes. Interestingly, in our differentiation condition, the barrier disruption co-occurred with the maturation of enterocytes, indicating a trade-off relationship (Fig. 4e,f). Intestinal epithelium is one of the organs with fastest turnover; intestinal stem cells divide at the crypt, migrate upwards to the villus while differentiating continuously, and finally shed from villus tips every 2–6 days^46^. Although the mechanism of cell shedding is still the subject of much discussion, the barrier disruption with enterocyte maturation in our differentiation condition might reflect the biological relationship between intestinal cell differentiation and cell apoptosis.

In conclusion, we developed a multi-functional monolayer culture as an in vitro model that can be utilized to assess the absorption of drug candidates regarded as P-gp or CYP3A substrates, validate in vitro–in vivo correlations, and evaluate drug-drug interactions. This tool might be used to better understand species differences in drug absorption between rats and humans, facilitating the development of highly absorbable drugs.

## Materials and methods

### Crypt isolation and organoid culture

Crypt isolation from RCCHan™: WIST female rat duodenum and organoid culture were conducted according to the previous report^21^. Crypts were isolated using Gentle Cell Dissociation Reagent (STEMCELL Technologies) for 30 min on ice. The digested contents were filtrated through a 70 µm strainer and centrifuged at 300×*g* for 5 min at 4 °C. The pellets were resuspended with ice-cold Advanced DMEM/F-12 and centrifuged at 150×*g* for 2 min at 4 °C. The crypts fraction was embedded with 100% Matrigel (growth factor reduced, Corning, Cat# 356231) in the basal medium, consisting of Advanced DMEM/F-12, penicillin/streptomycin (Thermo Fisher Scientific), 10 mM HEPES (Thermo Fisher Scientific), 2 mM GlutaMAX-I (Thermo Fisher Scientific) supplemented with 1 × B-27 Supplement (Thermo Fisher Scientific), 1 mM *N*-acetyl-cysteine, 10 nM Gastrin (Sigma-Aldrich), 100 ng/ml recombinant mouse Noggin (Peprotech), 50 ng/ml recombinant mouse EGF (Thermo Fisher Scientific), 100 ng/ml recombinant human IGF-1 (BioLegend), 50 ng/ml recombinant human basic FGF (FGF-2) (Reprocell), 1 µg/ml recombinant human R-spondin 1 (FUJIFILM Wako Pure Chemical), 500 nM A83-01 and 10% Afamin/Wnt3a CM (JSR). Budding type organoids were picked up and passaged by mechanical disruption. 10 µM Y-27632 was supplemented for the first 2 days of culture until the first passage, and EGF was removed from the first passage. Rat organoids were maintained in ΔE medium, consisting of basal medium supplemented with 1 × B-27 Supplement, 1 mM *N*-acetyl-cysteine, 10 nM Gastrin, 100 ng/ml Noggin, 100 ng/ml IGF-1, 50 ng/ml heat stable recombinant human bFGF (Thermo Fisher Scientific), 1 or 0.5 μg/ml R-spondin 1, 500 nM A83-01 and 10% Afamin/Wnt3a CM. For the monolayer culture, rat organoids were dissociated with TrypLE Select Enzyme (10×) for 5 min in a 37 °C water bath and filtered through a 70 µm cell strainer. The filtrate was centrifuged at 300×*g* for 3 min at 4 °C and supernatant was discarded, and the single cells were resuspended with ΔE medium supplemented with 50 ng/ml EGF and 10 µM Y-27632. 100 µL of cell suspension (7.0 × 10^4^ cells/well) was added into cell culture insert (Corning, Cat#353095) coated with Matrigel diluted 30-fold with PBS for at least 2 h at 37 °C. Y-27632 was supplemented with culture medium only on the first day. Bright-field images were obtained with an inverted microscope (IX83, Olympus).

### Development of cyst-enriched organoids

Rat organoids were dissociated with TrypLE Select Enzyme (10×) for 5 min in a 37 °C water bath and filtered through a 70 µm cell strainer. Single cells were resuspended with cyst-enriched medium consisting of basal medium supplemented with 1 × B-27 Supplement, 1 mM *N*-acetyl-cysteine, 10 nM Gastrin, 100 ng/ml Noggin, 50 ng/ml EGF, 100 ng/ml IGF-1, 50 ng/ml heat stable bFGF, 0.5 µg/ml R-spondin 1, 500 nM A83-01, 10 µM Y-27632 and 20% Afamin/Wnt3a CM. 3.0 × 10^4^ cells were embedded with 50 µl of Matrigel and cultured with cyst-enriched medium. The medium was replaced every 2 or 3 days, and cyst-enriched organoids were passaged every 3 or 4 days by mechanical dissociation.

### Monolayer culture

Conventional organoids/cyst-enriched organoids were dissociated into single cells using TrypLE Select Enzyme (10×). After a filtration by 70 µm cell strainer and centrifugation, the cell pellet was resuspended in cyst-enriched medium and adjusted to 3.5 × 10^5^ cells/mL. 200 µL of cell suspension was seeded on Transwell (Corning, Cat#3378 or 3470) coated with Matrigel diluted 50-fold with cold-PBS at 37 °C for 2 h, and medium was added to the basal compartment. On day 2 or 3, the medium was changed to the differentiation medium which consisted of basal medium containing 1 × B-27 Supplement, 1 mM *N*-acetyl-cysteine, 10 nM Gastrin, 100 ng/ml Noggin, 0.5 µg/ml R-spondin 1, 500 nM A83-01, 10 µM Y-27632, 3 mM VPA (Abcam). The medium was replaced every 2 or 3 days. For the induction assay, differentiation medium containing 50 µM β-naphtoflavone (Sigma), 5 µM 3-methylcholanthrene (Sigma), or 100 nM 1α, 25-dihydroxyvitamin D3 (Sigma) was exposed for 48 h. CYP1A activity and CYP3A activity were analyzed by P450-Glo Assays according the manufacturer’s protocol (luciferin-CEE for CYP1A, luciferin-IPA for CYP3A, Promega). The monolayer integrity was assessed by TEER. The TEER value was measured using Millicell ERS-2 system (Merck).

### Histological examination

A paraffin block of rat organoids were prepared by the method described previously^47^. Briefly, rat organoids were fixed with 4% Glutaraldehyde for 1 h at room temperature. Matrigel dome was disrupted by pipetting and collected, then embedded in paraffin by the AMeX method^48^. HE slides were prepared and an AB-PAS was performed.

### Whole mount immunostaining for organoids and monolayer

After withdrawing the culture medium, Matrigel dome was fixed by 2% paraformaldehyde (PFA) for 30 min at room temperature. After PBS washing, PBS containing 0.5% Triton X-100 was added and permeabilized at 4 °C overnight. Blocking was performed with BlockAid Blocking Solution (Thermo Fisher Scientific) containing 0.5% Triton X-100 for 6 h at room temperature. Primary antibodies [Alexa Fluor 555-labeled anti-Ki-67 antibody (Clone B56, BD Biosciences), anti-Sox9 antibody (Clone D8G8H, Cell Signaling Technology), anti-CK20 antibody (Clone SA35-03, Thermo Fisher Scientific), anti-P-gp antibody (Clone F4, Thermo Fisher Scientific), anti-ZO-1 antibody (Clone ZO1-1A12, Thermo Fisher Scientific), and anti-phospho-Ezrin antibody (Clone 48G2, Cell Signaling Technology)] in blocking solution containing 0.5% Triton X-100 were added and incubated for 3 days at 4 °C. After that, the sample were washed with PBS containing 0.5% Triton X-100 three times, incubated for 1 or 2 days at 4 °C with secondary antibody [Alexa Fluor 488-labeled anti-rabbit IgG antibody (Thermo Fisher Scientific), Alexa Fluor Plus 555-labeled anti-rabbit IgG antibody (Thermo Fisher Scientific), or Alexa Fluor 555-labeled anti-mouse IgG1 antibody (Thermo Fisher Scientific)] in blocking solution containing 0.5% Triton X-100. After washing with PBS containing 0.5% Triton X-100 three times, the wells were then incubated with SeeDB2G Solution 1 [1/3 × Omnipaque350 with 2% saponin] for 3 h at room temperature, Solution 2 (1/2 × Omnipaque350 with 2% saponin) containing Phalloidin-DyLight 650 and 1 µg/mL DAPI overnight at 4 °C, Solution 3 (1 × Omnipaque350) for 3 h at room temperature to clear the organoids^49^, which were observed with a confocal fluorescence microscope (A1, Nikon).

For the monolayer culture, after withdrawing the culture medium, the monolayer was fixed by 4% PFA for 15 min at room temperature. After PBS washing, blocking solution containing 0.5% Triton X-100 was added and incubated for 1 h at room temperature. The monolayer was stained with primary antibodies [anti-P-gp antibody, anti-ZO-1 antibody, anti-phospho-Ezrin antibody, anti-E cadherin (Thermo Fisher Scientific, PA5-32178), anti-Villin-1 antibody (Clone R814, Cell Signaling Technology)] in blocking solution containing 0.3% Triton X-100 at 4 °C for 2 days, then washed with PBS three times, incubated overnight at 4 °C with secondary antibody in blocking solution containing 0.3% Triton X-100.

### Transmission electron microscopy for organoids

Organoids were fixed with half Karnovsky solution at 4 °C for 24 h. Fixed organoids with Matrigel were minced into pieces of approximately 1 mm^3^ with a razor blade and washed with 0.1 M cacodylate buffer. Further fixation was performed using 1% osmium tetroxide in 0.1 M cacodylate buffer at 4 °C for 1.5 h. The fixed organoids were dehydrated while gradually increasing the ethanol concentration from 50 to 100%, replaced with propylene oxide, and embedded in epoxy resin (Quetol812, Nisshin EM). Resin was heat polymerized; ultrathin (60–70 nm) sections were prepared using a ultramicrotome (Leica EM UC7, Leica Microsystems), double-stained with uranyl acetate and lead citrate, and observed with a transmission electron microscope (HT7700, Hitachi High-Technologies).

### Organoid formation assay

Conventional organoids and cyst-enriched organoids were dissociated into single cells in the same way as the monolayer culture. These cells were embedded with Matrigel and cultured in expansion medium supplemented with 10 μM Y-27632 for 4 days. Organoid growth was measured using CellTiter-Glo^®^ 3D Cell Viability Assay (Promega) after solubilizing Matrigel with dispase (Dispase I, FUJIFILM Wako Pure Chemical). For the Imaging analysis was performed with cellSens (OLYMPUS) according the manufacturer. The formation of an organoid was defined to be when the major axis of its structure was measured to be over 100 μm in the bright-field image.

### EdU uptake assay

EdU staining was performed according the manufacturer’s protocol (Click-iT Plus EdU Cell Proliferation Kit for imaging, Alexa Fluor 555 dye, Thermo Fisher Scientific). Nuclei and EdU positive cells were counted using NIS elements (Nikon) according to the manufacturer’s instructions.

### Quantitative RT-PCR

Total RNA was extracted from monolayer culture using TRIzol. cDNA was synthesized with the Superscript III First-strand synthesis system for RT-PCR (Thermo Fisher Scientific). Quantitative RT-PCR was conducted in duplicate for each gene on the StepOnePlus Real-Time PCR System (Thermo Fisher Scientific) using SYBR Green (Thermo Fisher Scientific). β*-Actin* was used as the housekeeping gene. The sequences of the primers for RT-PCR are shown in Supplementary Table S1.

### Compound permeability assay

For the lucifer yellow permeability assay, a transporter buffer consisting of 10 mM HEPES and 1% (w/v) BSA in HBSS (pH 7.4) with 200 μM lucifer yellow was added to the apical side. HBSS was purchased from Thermo Fisher Scientific (Cat#14025092). The transporter buffer without lucifer yellow was also added to the basal side and cultured by shaking. After 2 h, the basal buffer was sampled for analysis. Lucifer yellow concentration was determined by a fluorescent signal measured with EnSpire (PerkinElmer) using 428 nm excitation and 536 nm emission filters. For the digoxin permeability assay, a transporter buffer containing 200 μM lucifer yellow and 10 μM digoxin in the presence or absence of 5 μM Zosuqudar was added to the apical or basal side, respectively. After 2 h, the basal or apical buffer was sampled for analysis. For the midazolam metabolic assay, HBSS containing 200 μM lucifer yellow, 5 μM midazolam, 10 mM MES, and 1% (w/v) BSA (pH 6.5) in the presence or absence of 1 mM ABT was added to the apical side of the differentiated monolayer. Transporter buffer consisting of 10 mM HEPES and 4% (w/v) BSA in HBSS (pH 7.4) was added to basal side of the monolayer. After 2 h, the basal buffer was sampled for analysis. The monolayer which was cultured for 3 days in expansion medium and for 4 days in differentiated medium with 100 nM VD3 induction for 48 h was used for the midazolam assay. The concentration of digoxin, and 1-hydroxymidazolam were determined by LC–MS/MS analysis. The apparent permeability coefficient (Papp) was calculated according to following equation:

$$Papp= \frac{dQ}{dt} \times \frac{1}{A\times {C}_{0}},$$

where dQ/dt represent compound transported to the basal or apical side per unit time, A is the surface area of the cell culture insert, and C_0_ is the initial concentration of compound.

### LC–MS/MS analysis

The collected buffer samples were mixed with methanol/acetonitrile (1/1, v/v) containing an internal standard (*d3*-digoxin for digoxin analysis, *d4*-1-hydroxymidazolam for 1-hydroxymidazolam). After centrifugation or filtration, supernatant was analyzed using the Nexera X3 system (Shimadzu), QTRAP6500 system (SCIEX), and the ACQUITY UPLC BEH C18 column (1.7 μm, 2.1 mm × 50 mm, Waters) for digoxin, or the XBridge BEH C18 Column (3.5 μm, 2.1 mm × 100 mm, Waters) for 1-hydroxymidazolam.

### Statistical analysis

Statistical analyses were performed using the GraphPad Prism 7.04 (GraphPad Software) by Student t-test and Dunnett’s multiple comparison test. A p-value of < 0.05 was considered statistically significant.

## Supplementary Information

Supplementary Information.

## Data Availability

All data generated or analyzed during this study are included in this published article.

## Abbreviations

3MC: 3-Methylcholanthrene
AB-PAS: Alcian blue-periodic acid-Schiff
ABT: 1-Aminobenzotriazole
ADME: Absorption, distribution, metabolism, and excretion
AhR: Aryl hydrocarbon receptor
bNF: Beta-naphthoflavone
CYP450: Cytochrome P450
DMPK: Drug metabolism and pharmacokinetics
EGF: Epidermal growth factor
Papp: Apparent permeability coefficient
P-gp: P-glycoprotein
PFA: Paraformaldehyde
Si: Sucrase-isomaltase
TEER: Transepithelial electrical resistance
VD3: 1Alpha, 25-dihydroxyvitamin D3
VDR: Vitamin D receptor
VPA: Valproic acid
