# Supplementary material for: Development of rat duodenal monolayer model with effective barrier function from rat organoids for ADME assay
Source: Sci Rep. 2023 Jul 26;13:12130. doi: 10.1038/s41598-023-39425-7 (PMC10372144; doi:10.1038/s41598-023-39425-7)
Supplement: Supplementary file 1 — Supplementary Information. [file 41598_2023_39425_MOESM1_ESM.pdf]

Supplemental Information

Development of rat duodenal monolayer model with effective barrier function from rat organoids for ADME assay

Authors

Kai Tanaka[1*], Shigeto Kawai[1], Etsuko Fujii[2], Masumi Yano[2], Takashi Miyayama[2], Kiyotaka Nakano[1], Kimio Terao[3], Masami Suzuki[4]

Affiliations

[1]Translational Research Division, Chugai Pharmaceutical Co., Ltd., 5-1-1 Tsukiji Chuo-ku, Tokyo 104-0045, Japan. [2]Translational Research Division, Chugai Pharmaceutical Co., Ltd., 216 Totsuka Totsuka-ku Yokohama, Kanagawa 244-8602, Japan. [3]Translational Research Division, Chugai Pharmaceutical Co., Ltd., 2-1-1 Nihonbashi-Muromachi Chuo-ku, Tokyo 103-8324, Japan. [4]Chugai Pharmaceutical Co., Ltd., 1-135 Komakado, Gotemba, Shizuoka 412-8513, Japan
*email: tanaka.kai11@chugai-pharm.co.jp

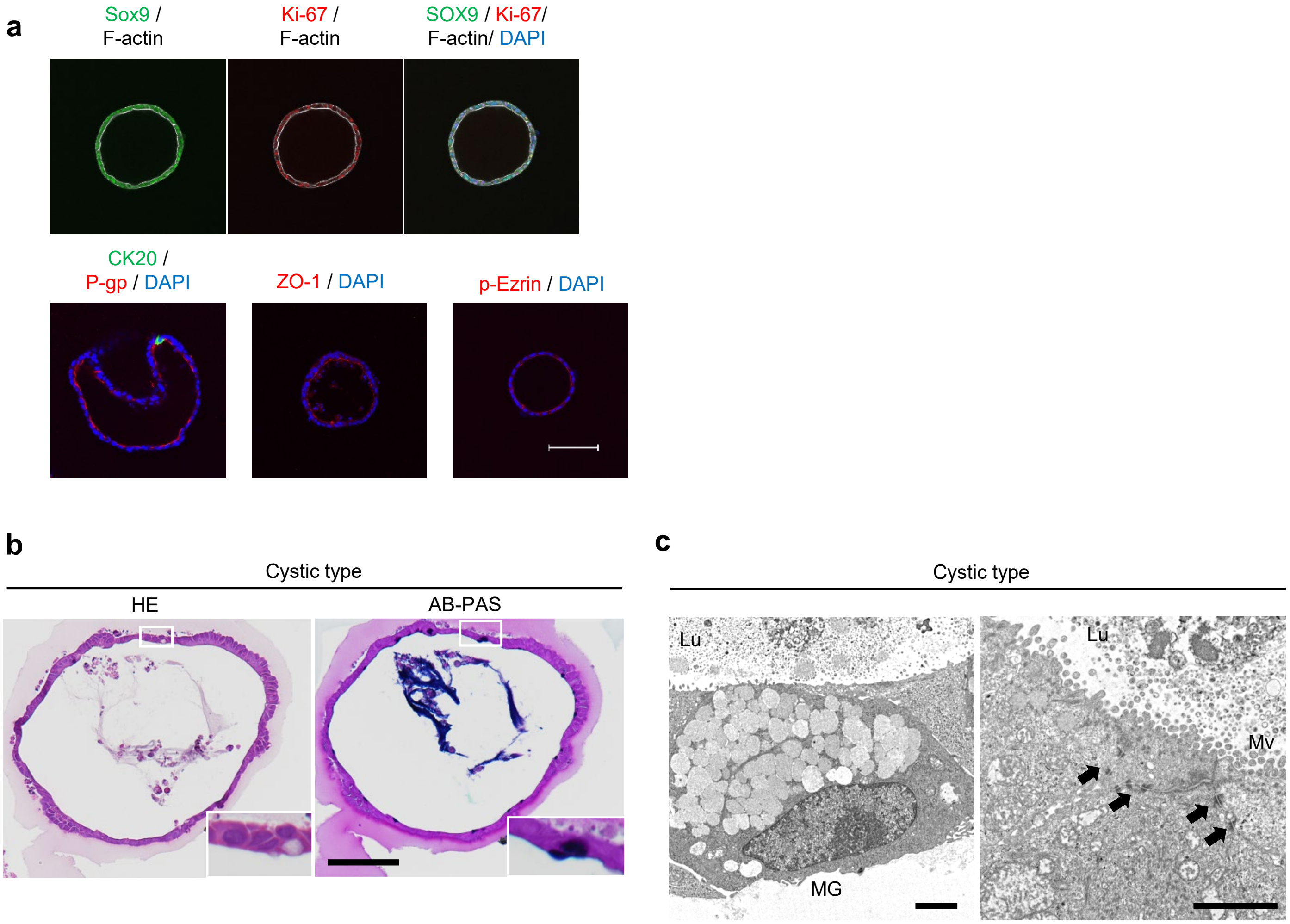

Supplemental Figure S1.
Characterization of rat duodenal organoid of cystic type. (a) Whole mount immunostaining of cystic type for Sox9 (green) and F-actin (white), Ki-67 (red) and F-actin (white), merge with DAPI (blue), CK20 (green) and P-gp (red) with DAPI (blue), ZO-1 (red) and DAPI (blue), phospho-Ezrin (p-Ezrin, red) and DAPI (blue). Scale bar = 100 μm (b) Day 4 organoids sections were stained with HE, AB-PAS. Scale bar = 100 μm. (c) Transmission electron microscopy of organoids on day 4. Arrows, desmosomes; MG, Matrigel; Mv, microvilli; Lu, Lumen. Scale bar = 2.0 μm.

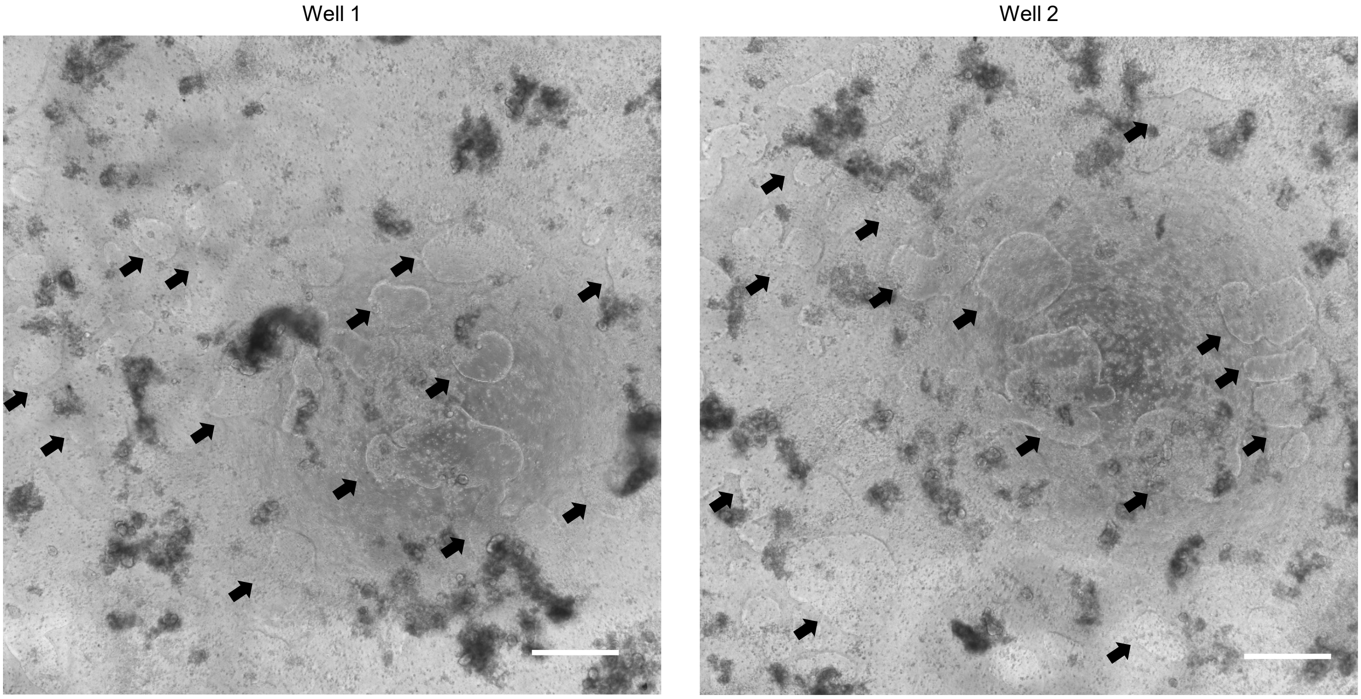

Supplemental Figure S2.
Day 7 monolayer culture generated by conventional rat organoids. Arrows show the cell absence area.
Scale bar = 500 μm

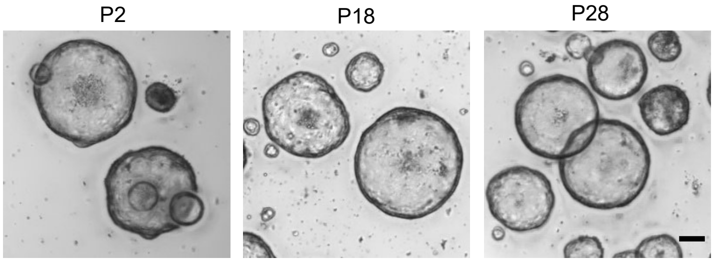

Supplemental Figure S3.
Representative images of long-term cultured cyst-enriched organoids.
Scale bar = 100 μm

Supplemental Table S1. Primers used for quantitative RT-PCR analysis

Gene	Forward primer	Reverse primer
Si	TGTTCCTAAGGCTCGATGGTTTG	TTAGATGCGTGTTCCGTGCTG
Abcb1a	AGATCGTGAAGGCAGCCAAG	AGAAGTAAGATGTGAGGCTGTCTGA
Abcb1b	TGACTCGGGAGCAGAAGTTTGA	GGCGAAGGTGATCCCAAAGA
Abcc2	TGAGCACCAGCAGCGATTTC	GAAGACGACCAAGTTTCCAACCA
Cyp1a1	GTCCCGGATGTGGCCCTTCTCAAA	TAACTCTTCCCTGGATGCCTTCAA
Cyp3a9	TTGTACGATGGTCGACAGCC	CCACTGGACCAAAGTTCCGT
Act-b	GGAGATTACTGCCCTGGCTCCTA	GACTCATCGTACTCCTGCTTGCTG
